# Advancements in Sustainable Plant-Based Alternatives: Exploring Proteins, Fats, and Manufacturing Challenges in Alternative Meat Production

**DOI:** 10.4014/jmb.2312.12049

**Published:** 2024-02-19

**Authors:** Minju Jung, YouKyeong Lee, Sung Ok Han, Jeong Eun Hyeon

**Affiliations:** 1 Department of Food Science and Biotechnology, College of Knowledge-Based Services Engineering, Sungshin Women's University, Seoul 01133, Republic of Korea; 2 Department of Next Generation Applied Sciences, Graduate School, Sungshin Women's University, Seoul 01133, Republic of Korea; 3 Department of Biotechnology, Korea University, Seoul 02841, Republic of Korea

**Keywords:** Sustainable food, alternative meat, plant-based proteins, fat substitutes, fat mimetics, hamburger patties

## Abstract

The rise in plant-based food consumption is propelled by concerns for sustainability, personal beliefs, and a focus on healthy dietary habits. This trend, particularly in alternative meat, has attracted attention from specialized brands and eco-friendly food companies, leading to increased interest in plant-based alternatives. The dominant plant-based proteins, derived mainly from legumes, include soy protein isolates, which significantly impact sensory factors. In the realm of plant-based fats, substitutes are categorized into fat substitutes based on fats and fat mimetics based on proteins and carbohydrates. The production of these fats, utilizing gums, emulsions, gels, and additives, explores characteristics influencing the appearance, texture, flavor, and storage stability of final plant-based products. Analysis of plant-based proteins and fats in hamburger patties provides insights into manufacturing methods and raw materials used by leading alternative meat companies. However, challenges persist, such as replicating meat's marbling characteristic and addressing safety considerations in terms of potential allergy induction and nutritional supplementation. To enhance functionality and develop customized plant-based foods, it is essential to explore optimal combinations of various raw materials and develop new plant-based proteins and fat separation.

## 1. Introduction

Emerging needs for alternative foods include the promotion of a sustainable environment for future food security and the accommodation of personal or religious preferences. The growing interest in healthy eating habits has led to increased consumption of plant-based foods and a continued rise in the vegetarian population. Within the realm of vegan dishes, plant-based meat substitutes, which are substitutes for processed animal meat products, are the most popular [1]. Vegetable meat substitutes are manufactured by combining protein-rich plant ingredients, such as soy protein, with auxiliary ingredients, including vegetable oil, seasonings, and quality enhancers [2]. Plant-based meat substitutes have been demonstrated to be relatively safe, as they are produced commonly used in general meat products or foods [3]. Due to differences in taste and flavor from existing animal meat, some vegetarian consumers have considered soy meat as meat alternatives. Recently, products that minimize sensory differences have been released, and consumer interest in plant-based foods continues to grow [4]. Many food companies, along with meat alternative brands and eco-friendly food companies, are entering the meat alternative business. It has been reported that, through recent technological advancements, some plant-based meats have succeeded in catching up with the taste of existing meat to a certain level [5]. Research institutions and businesses are investing in plant-based foods to commercialize a variety of vegetable proteins and vegetable fats. As a result, numerous patent applications for different materials and processing methods have been filed [6].

In-depth research on the components of vegetable protein and fats makes the creation of meat substitutes conceivable. A study is currently exploring crop-derived protein and fat materials with a similar taste and texture to animal meat [7]. Depending on the type of vegetable protein used, manufacturing technology, and added additives, a variety of meat substitutes can be produced. This is due to the fact that the final alternative meat product’s quality, such as texture and flavor, varies depending on the type and composition of vegetable protein and fat [8]. In addition, meat alternatives have the ability to combine various nutrients in optimal ratios, making it easy to control their nutritional content through the selection of raw materials. Based on these characteristics, it is possible to produce personalized products, which allows for high market competitiveness [9]. Accordingly, it is important to understand the characteristics of vegetable proteins and fats used in meat alternatives and to utilize their pros and cons well.

**Fig. 1:**
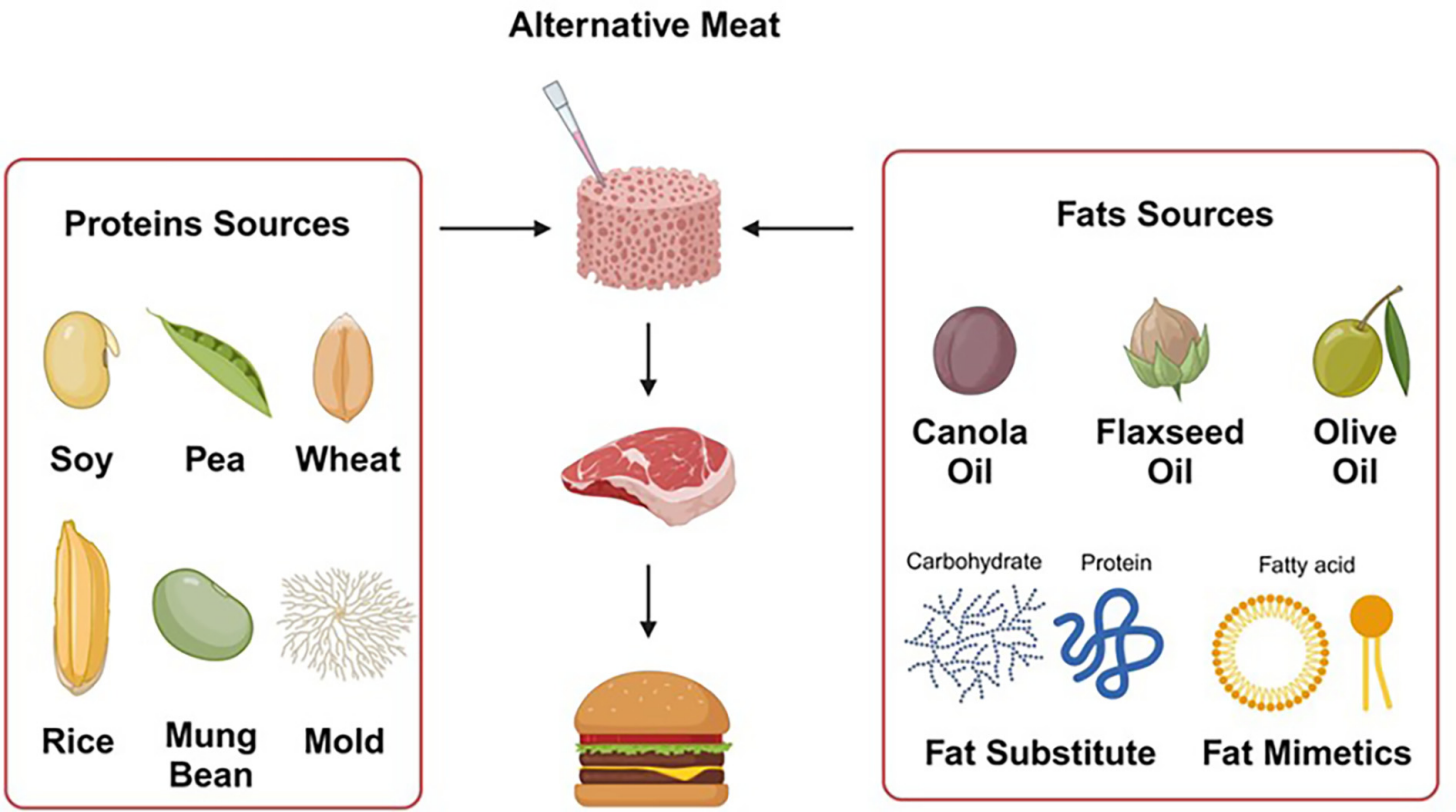
The scheme of vegetable proteins and fats used in meat alternatives.

While numerous studies have explored vegetable proteins and fats, there remains a gap in research concerning their quality [10]. Therefore, this study aims to analyze: 1) the development technology of representative vegetable proteins and vegetable fats that have recently been used as raw materials for meat alternatives; and 2) the physicochemical and functional properties of vegetable proteins and fats to determine their impact on the processing quality of alternative meat. We organized the content of vegetable proteins and fats and the applied technology of the various types of commonly mentioned plant-based foods (Fig. 1). As an additional scope, we confirmed the characteristics that distinguish plant-based food ingredients from their animal-based counterparts. Furthermore, we summarize technological advancements considering the current market situation and plant-based alternative foods that have been developed to improve accessibility, health, and preference with rich nutrients and flavor. Due to the low consumer preference for plant-based meat substitutes, the development of plant-based food materials is slow, and research for commercialization is still minimal. Thus, we would like to make suggestions for future development and growth so that plant-based meat substitute raw materials can contribute to the global market.

## Vegetable Protein Sources and Development Technology

Vegetable protein is produced by processing the defatted material of oil-pressing seeds (*e.g.*, soybeans) and grain powder (*e.g.*, wheat) to enhance the protein content [11]. Specific functions such as gel formation, emulsification, and other physical properties are created by heating or applying pressure. Vegetable proteins used in meat alternatives include soy protein, pea protein, and wheat protein as well as seeds such as canola and sunflower seeds, peanuts, rice, and mung bean proteins [2]. Vegetable protein materials are lower in calories compared to animal-based materials and contain various bioactive substances, such as polyphenols, that are not found in animal-based materials [12]. Vegetable proteins, which are beneficial for intestinal health due to their low fat and cholesterol contents, low calories, and rich dietary fiber and antioxidants, are not only used as meat alternatives but also in various beverages [13]. Each vegetable protein has a specific water absorption capacity, oil absorption capacity, emulsification activity, emulsion stability, water-soluble nitrogen index, DPPH (2,2-diphenyl-1-picrylhydrazyl) radical scavenging activity, and total polyphenol content [14]. Therefore, the final quality of plant-based protein foods varies depending on the type of plant-based protein used.

### Vegetable Protein Sources

Plant-based meat substitutes are meat-like products manufactured using proteins extracted from plants. soy protein, pea protein, wheat gluten, rice protein, mung bean protein, and mold are used as main raw materials [15]. Vegetable proteins are primarily derived from various legumes, with textured soy protein constituting the largest share in the meat substitute food market [16]. Extruding powder or concentrate containing at least 50% protein at high temperatures and pressure produces soy protein, which exhibits meat-like qualities. Soy protein has several advantages, such as high water holding capacity and gelation induction, making it suitable for texturizing [17]. Peas, which do not cause allergies and have a unique nutritional composition, are also attracting attention. In addition to soy and pea protein isolates, there are also wheat and rice proteins (Table 1).

### Soy proteins

Soy protein, a vegetable protein, is effective in weight loss through appetite control and helps improve metabolic syndromes such as cardiovascular disease [18]. Meat-like products made from soy protein have the advantages of being cholesterol-free, low in fat, and low in heat energy. For its beneficial nature, processing technology using soybeans has been continuously developed [19]. Soy protein is a high-quality protein source with a good balance of essential amino acids. It is not only beneficial to your health but also has the advantage of being environmentally friendly [20]. It is mainly used to increase protein content in snacks and beverages and can be applied to various types of foods. Soy protein, which can replace animal protein in processed meat and seafood products, improves product quality economically while maintaining the original texture. Soy protein isolate, which consists of 90%protein, has a high digestibility and is most often used as a replacement or supplement to animal protein [20].

### Pea proteins

Peas are rich in essential amino acids, and 20% of peas are protein. Globulins constitute 65%–80% of pea protein, while albumins constitute 10%–20% [21]. It exhibits functional properties similar to soy proteins and does not cause allergies like rice proteins [22]. One report stated that the product characteristics would be similar even if pea protein isolate were added instead of milk protein [23].

### Wheat proteins

Tivall, manufactured in Israel, is a meat alternative made from wheat protein. This product is manufactured by mixing wheat gluten and vegetable proteins [24]. Additionally, it is known to have good taste and nutrition and to be hygienically stable [25]. A meat-like texture is created through the texturizing process, and other minerals are mixed depending on the desired final product. It is mainly used in the production of burgers, sausages, veal cutlets, and nuggets.

### Rice proteins

Rice protein has a relatively high content of essential amino acids among vegetable proteins, has a balanced composition, and has been reported to be not only nutritionally excellent but also exhibit various physiological activities such as antioxidant and anticancer effects [26]. Rice protein, which has recently been drawing attention as an immunity-boosting protein supplement, appears to be a useful source for people with immune diseases due to its low incidence of allergies [27]. It is suitable for foods that do not require high solubility, and hydrolyzed rice protein can be used as a functional source. Since rice contains little lysine, it is possible to manufacture more effective products by combining it with lysine-rich soy isolate or pea isolate protein.

### Mung bean proteins

Mung beans, known for their resilience to drought, are regarded as "green pearls" due to their high protein content (25-28%) and low-fat content (12%) [28]. The abundant presence of proline, glutamic acid, arginine, leucine, and phenylalanine has led to the isolation of high-quality protein from mung beans. Mung beans exhibit detoxification activity, alleviate cardiovascular diseases, and regulate gastrointestinal disorders [29]. Recent evidence has proven that mung beans possess properties such as lowering blood sugar, anti-melanogenic effects, immune regulation, and liver protection, surpassing fundamental bodily nutritional requirements [30]. When appropriately processed, mung beans can serve as a cost-effective protein source in the form of textured plant-based protein, benefiting vegetarian consumers [31].

### Mold proteins

In recent studies, attempts have been made to develop burger patties using fungal protein [32, 33]. Generally, these patties boast a higher protein content compared to traditional hamburger products. Notably, their nutrient composition stands out among competitors in the market. This innovative approach to incorporating fungal protein into burger patties presents a promising avenue for creating nutritious and protein-rich alternatives in the realm of plant-based foods. Further exploration of this emerging technology may lead to novel and sustainable solutions for the growing demand for alternative protein sources.

### Features of Development Technology for Vegetable Proteins

Spinning, extrusion molding, and steaming are manufacturing technologies and production processes that generate a texture similar to that of animal protein, with extrusion molding being the most representative. The extrusion process gained prominence in the production of texturized protein products, specifically plant-based meat substitutes. This method utilized defatted soybeans and concentrated soybean protein as raw materials, reflecting a growing belief that vegetable protein could serve as a viable replacement for animal protein [34]. This process allows for manufacturing with excellent quality in terms of texture, resulting in a more meat-like texture. One of the products that is receiving a lot of attention is the “Impossible Burger.” It uses various flavor precursors and leghemoglobin extracted from soybeans to mimic the sensory characteristics of beef [35].

Continuous efforts are being made to improve the palatability of plant-based meat alternatives, such as their appearance, flavor, texture, and ability to match that of meat. It must have a structure or tissue similar to that of animal muscle and must also have functional properties such as water retention capacity and fat adsorption [36]. All flavors, spices, and coloring substances added during manufacturing must be continuously retained within the tissue, even during cooking. Therefore, meat alternatives can be manufactured in a variety of products depending on the type of vegetable protein used, the production technology for texturizing the vegetable protein, and the addition of additives. The type and content of plant proteins affect the physicochemical and functional properties of the final product [2]. In each combination of vegetable protein additions, isolated soy protein acts as a factor that has the greatest effect on overall preference, such as appearance and texture, and the higher the addition ratio of soy protein isolate, the higher the preference [37]. Pea protein exerts the greatest influence on flavor, and as the addition ratio increases, so does consumer preference [6]. Rice protein has a significant effect on moisture content and color, and as the addition ratio increases, moisture content and color are found to increase [38]. Soy protein isolate has the highest concentration of crude protein. This was followed by wheat protein, pea protein isolate, and rice protein. However, the crude protein content of the four main protein ingredients listed above, including soy protein isolate, was less than 1% [20]. The scavenging activity of DPPH is a standard of the antioxidant effect of the extract. The polyphenol compound content and DPPH radical scavenging activity are in the following order: pea protein, rice protein, wheat protein, and soy isolate [14]. In cases of following a diet with an increased intake of plant-based foods such as fruits, vegetables, beans, whole grains, nuts, and vegetable oils to reduce the risk of metabolic diseases, the above four vegetable protein sources are the most commonly known [39]. If these four types of plant proteins are combined well, the manufactured product can have the same flavor as meat while also promoting health, particularly in the case of cardiovascular disease.

## Vegetable Fat Sources and Development Technology

Regarding plant-based meat alternatives, research is needed not only on plant protein materials but also on plant lipids. Vegetable fat refers to the oil contained in the fruits or seeds of plants, such as cocoa butter, sorghum fat, and shea butter. Vegetable fats rich in unsaturated fatty acids are known to help lower blood cholesterol [40]. The use of vegetable oils with low trans fats to replace animal fats is increasing, and related research is also being actively conducted [41]. Animal fat is known to play an important role in the taste, aroma, and physical properties of meat products. When developing plant-based foods, it is desirable to use vegetable fats that can maintain the unique texture, flavor, and appearance of animal fats [15]. Compared to research on vegetable proteins, there is a relatively insufficient amount of research on vegetable fats. Therefore, in this review, our aim is to examine the use of vegetable oil as a substitute for animal fat in meat alternatives and explore traditional fat replacers.

### Vegetable Fat Sources

**Fig. 2:**
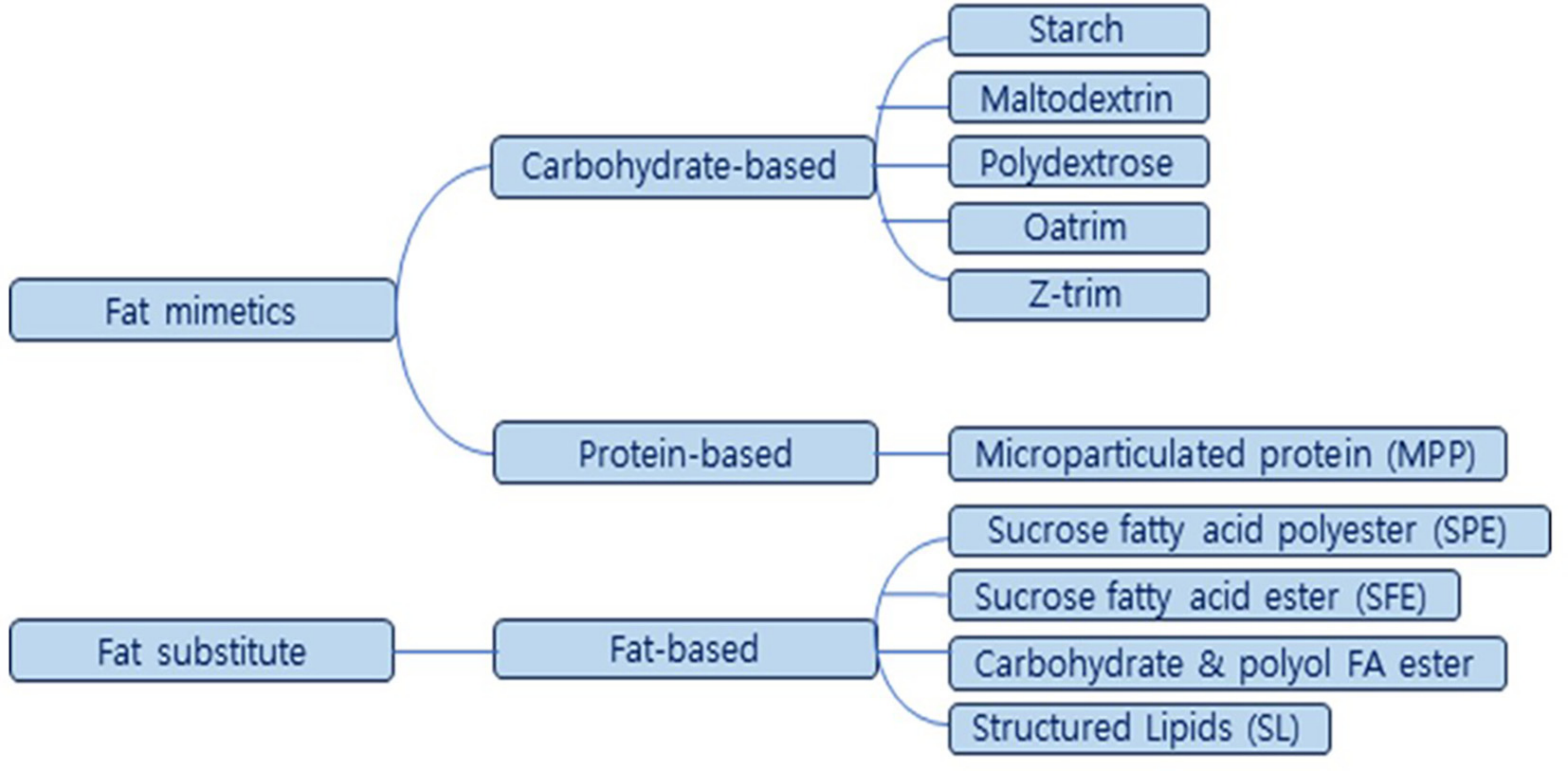
Two main categories of fat replacers: fat mimetics based on carbohydrates and proteins, and fat substitutes based on fats and those.

Vegetable oils, such as medium-chain triglycerides (MCT) oil and canola oil, are commonly utilized as vegetable fats. These vegetable oils are utilized in a refined form with a high content, offering advantages such as water-holding capacity, gelation induction, and emulsion stability. This confers the advantage of achieving a texture similar to traditional meat when producing meat alternatives [17]. Other than vegetable oil, there are two main categories of fat replacers: those based on fats and those based on proteins and carbohydrates (Fig. 2). ‘Fat replacer’ is a broad term commonly used to describe both types [42]. The category of fat mimetics includes substances that imitate the physical or taste-related properties of fat, particularly in products with high moisture content. Examples of fat mimetics are carbohydrates and proteins. Another category is fat substitutes, which are triglycerides that have been physically and chemically reconstituted. These aim to provide all the functional properties of fat while asserting to be low in calories. This category also includes fat substitutes derived directly from fats.

### Vegetable Oils

MCT oil, primarily converted into an energy source upon absorption into the body, is utilized for dietary and therapeutic purposes. Additionally, it helps in preserving the flavor components in the emulsion state [43]. Flaxseed oil and chia seed oil are effective in reducing arteriosclerosis and blood clot formation and contain more than 0.85% of saturated fatty acids compared to unsaturated fatty acids [44]. Canola oil shows excellent quality as a fat substitute in terms of tenderness, juiciness, and overall palatability [45]. Additionally, sunflower oil promotes the production of phenolic compounds and enhances nutritional properties [8]. Olive oil is rich in unsaturated fatty acids, including oleic acid, and reduces the risk of cardiovascular diseases [46]. Other vegetable oils, such as castor oil, orange oil, palm oil, shortening, and margarine, have been added to meat substitutes [47]. Meat analogues with orange oil had high moisture content, relatively high liquid retention capacity, and high DPPH radical scavenging activity regardless of the amount of oil added. Sensory evaluation also showed that the smell of soybeans decreased, and the juiciness increased. The addition of orange oil improved the preference and quality of meat analogues by increasing meat juiciness and reducing the soybean smell in the product [47]. In addition, garlic inulin was utilized as a fat substitute in the production of low-fat chicken sausages. Its widespread application includes the use of cold-pressed pumpkin seed oil as a replacement for beef fat in the manufacturing of Bologna-type sausages, canola oil as a solid fat substitute in plant-based cheese, and carrots and lemon as fat substitutes for enhancing the physicochemical, textural, and sensory qualities of low-fat beef burgers. Fibers are also incorporated in these applications [48-50].

### Fat Replacer : Fat Mimetics

Carbohydrate-based fat mimetics, including gums, starch, pectin, cellulose, corn syrup, and polyol, are predominantly recognized as Generally Recognized as Safe (GRAS) substances and have been utilized as thickening agents and stabilizers since ancient times [51]. There are over 40 varieties of starch-based alternative fats, employing both native and modified starch in pregelatinized or instant forms. Notably, they offer the advantage of preventing the hardening phenomenon commonly observed when using items like bread and cookies in a microwave oven. Maltodextrin, produced by hydrolyzing corn starch with acid or enzymes, finds application in sauces and salad dressings. Polydextrose, a glucose, sorbitol, and citric acid polymer, is available as Pfizer's 'Litesse.' Oatrim, derived from partially hydrolyzing the starch-containing part of oat bran and outer skin with enzymes, is marketed as Staley's 'TrimChoice.' Z-trim, developed by the USDA G.C. Inglett, is a non-digestible, insoluble fiber sourced from high-fiber components of soybeans, soybeans, rice, and the bran of corn and wheat. Inulin, being an indigestible carbohydrate, serves as a prebiotic and can function as a fat substitute. Therefore, it is employed either independently or in conjunction with other relevant ingredients within the food industry, serving various purposes such as a dietary fiber, low-calorie sweetener, fat substitute, gelling agent, viscosity modifier, and texture-modifying ingredient across a range of foods.

Protein-based fat mimetics refer to microparticulated protein products (MPP) created by shaping proteins from egg, milk, whey, soybean, and wheat into small, spherical particles (50 billion/1 tsp; diameter 1~15 μm) [52]. The diminutive size of these particles imparts a creamy taste and texture in the mouth, and they exhibit freeze-thaw stability. NutraSweet's Simplessse is recognized as the pioneer among alternative fats in this category.

### Fat Replacer: Fat Substitutes

Sucrose fatty acid polyester (SPE) relies on the strong bonding between the hydroxyl group of sugar and the carboxyl group of fatty acid. It is produced by forming ester bonds with 6 to 8 fatty acids in a single sugar molecule [53]. The physical properties, functionality, and usability of SPE are ultimately determined by the type and form of the combined fatty acid. A notable example is Procter & Gamble's Olestra, derived from fatty acids found in vegetable oils like soybean oil, corn oil, and cottonseed oil. Sucrose fatty acid ester (SFE) exhibits outstanding emulsifying properties, making it useful as an emulsifier and stabilizer [54]. Carbohydrate fatty acid ester and polyol fatty acid ester are alternative fats formed through ester bonding with fatty acids and polyols containing 4 or more hydroxyl groups, such as sorbitol, trehalose, raffinose, and stachyose [42]. Structured lipids are triglycerides containing high, intermediate, and low fatty acids, synthesized through chemical methods or enzymes, or created via random transesterification. With a broad range of applicable fatty acids and a wide melting point range, structured lipids can be employed in a diverse array of food products. In dairy products, various fat substitutes are employed, including carbohydrate-based cellulose, gums, inulin, maltodextrins, maltose, oatrim, polydextrose, starches, as well as protein-based microparticulated protein, modified whey protein concentrate, and fat-based emulsifiers like olestra. In the case of meat and poultry, fat substitutes consist of carbohydrate-based gums, inulin, maltodextrins, oatrim, polydextrose, and starches [52].

### Features of Development Technology for Vegetable Fats

Replacing animal fat with vegetable oil decreases the hardness of hamburger patties or sausages. Elasticity, gumminess, and chewiness tend to decrease along with hardness. Additionally, the change in patty thickness in the vegetable oil-treated group is small. Previous research results also showed that low-fat patties partially containing various vegetable oils have a significantly lower thickness reduction rate than regular patties [55]. Additionally, the patty color is influenced by the type and concentration of fat used. Sausages made with vegetable oil had higher brightness values than sausages made with pork fat, and this result was confirmed in later studies [56]. The difference in color parameters is because small droplets of vegetable oil reflect more light than large droplets of animal fat [57]. In addition, the results of a study on the quality and sensory evaluation of madeleines using vegetable oil confirmed the same result that when replacing animal ingredients with vegetable oil and soy milk, madeleines were produced with a brighter color than when using animal ingredients [58].

Physical property tests showed that the hardness, cohesiveness, adhesiveness, and chewiness values were significantly reduced in products mixed with lecithin. Sensory testing using principal component analysis showed that overall preference increased as the juiciness and softness of the sample increased [59]. It was confirmed that the sample with added emulsion had higher values in terms of juiciness, softness, and overall preference compared to other samples. Partial least squares regression analysis was performed to examine the relationship between samples for sensory tests and physicochemical properties. The juiciness and softness of the samples increased as the heat loss increased. It was confirmed that hardness, cohesiveness, elasticity, adhesiveness, and chewiness, excluding adhesion, had a positive correlation with liquid holding capacity and showed an opposite trend to the juiciness and softness of the samples [60]. Fats-derived substitutes can be formulated by combining short- or medium-chain fatty acids while preserving the triglyceride structure. Alternatively, they may be newly synthesized to achieve "0" calories and can express their functionality by effectively utilizing emulsifiers. Fat substitutes play diverse roles in foods, such as retaining moisture, delaying aging, enhancing texture and physical properties, ensuring emulsion stability, and reducing oil absorption [61].

## Utilization of Vegetable Protein and Vegetable Fat in Hamburger Patties

The Impossible Burger's patty contains protein, vitamins, sugar, amino acids, and konjac from wheat flour and potatoes, while coconut oil and soybeans are used to replicate the fat content of meat [8]. When grilling the patty, oil flows out just like when grilling actual meat, resulting not only in a delicious taste but also in a meat-like cooking experience. The Impossible Burger was created by extracting heme from soy leghemoglobin and culturing it to achieve its red color and juiciness. Beyond Meat, on the other hand, utilized coconut and olive oil to recreate juiciness, extracting vegetable protein from beans, mushrooms, pumpkins, etc. Beets were used to achieve a red color, and they were cultured with yeast and fiber to reproduce the flavor and texture of meat. Beyond Meat distinguishes itself by using peas, not soybeans, as its main ingredient. Additionally, in products such as sausages, a combination of peas, yams, soybeans, and rice proteins were used to achieve a sausage-like texture, while beets were employed to reproduce an appetizing red color. Italy's Nuova Due introduced the Porcini mushroom burger, and various methods for developing hamburger patties have emerged, such as Germany's LikeMeat with their Juicy burgers made from legume patties. Various burger mixes are also available for purchase. The primary vegetable proteins employed in alternative meat production include β-conglycinin, gluten, glycinin, vicilin, legumin, albumins, globulins, and glutelins. The fats used in vegetable substitute meat products are predominantly vegetable oils, with MCT oil being the main choice [62]. This oil is absorbed into the body and serves primarily as an energy source, making it effective for patients and modern individuals on a diet. In an emulsion state, it plays a role in reducing the loss of flavor components. In addition to burger patties, plant-based food options have expanded to include meatballs, nuggets, pork cutlets, chicken tenders, bulgogi, and ham.

As research and development progress to analyze the composition and texture of real meat at the molecular level, each company is also developing recipes that adjust the combination of raw materials and temperature settings (Table 2). Vegetable oil is added to plant-based meat substitutes, including hamburger patties, because fat surrounds and softens sticky proteins [63]. Olive oil and canola oil are also used, but coconut oil, which is solid at room temperature, is typically preferred. Coconut oil, being solid at room temperature with a distinct melting point, provides a mouthfeel similar to animal fat, allowing major patty manufacturers to leverage it to bring out the characteristics of beef fat. However, unlike beef fat, which melts at 40-50°C, coconut oil melts at 25°C, making it challenging to perfectly replicate the mouthfeel texture.

## Market Status for Vegetable Proteins and Fats

Plant-based food consumers are broadly classified into three groups. The first are vegetarians who abstain from meat products due to religious beliefs. Grains, vegetables, fruits, nuts, etc. are their daily diet. The second are consumers looking for health-oriented foods that can replace meat. They are constantly interested in their health to prevent metabolic syndrome and try to intake nutrients lacking in meat through plant-based foods. The third group are consumers looking for a more affordable protein source. They are looking for meat substitutes due to the rise in meat prices caused by a limited meat supply. Most meat substitutes are meat supplements made from vegetable proteins, including soybeans, and vegetable fats, such as ham and hamburgers [64].

The development of plant-based meat using soy protein has been widely developed overseas in the form of patties, sausages, and meat alternatives by companies such as Impossible Foods, Beyond Meat, and Hampton Creek. According to the report on market trend analysis for fat replacers, the fat replacer market in the US is expected to grow based on the data in 2014 [9]. According to the global hydrocolloid industry trend report (Data Bridge Market Research), the European market for fat replacer is expected to grow to USD 3,495.56 million by 2028. It is predicted that Germany, the UK, France, and Belgium would be the main axes of development. According to the fat replacer market trend analysis report (SkyQuest Technology Group), fat replacers are most often applied to bakery and confectionery products, followed by beverages, convenience foods, dressings, margarine and spreads, processed meat, dairy products, and frozen desserts in order. The bakery sector is expected to show the highest growth rate by 2028, and the growth of the food and beverage sector in the Middle East and BRICs (Brazil, Russia, India, China, etc.) countries is expected to be a major driving force in the market. In particular, the increasing global consumption of skim milk is one of the key factors driving the growth of the fat-replacer beverage market. A survey by the Calorie Control Council found that 88% of adults prefer low-fat or fat-free foods and beverages [14]. Cargill's Olinera and Milfar are the products of global companies that produce materials and products overseas. As fat replacers are widely used in the confectionery field, Olinera, a cocoa butter replacer, maintains the gloss of chocolate products, crystallizes quickly at low viscosity, and shows excellent elasticity even at thin thickness, preventing coating cracks. Additionally, Milfar, a milk fat replacer, is used in cheese and bakery products to create a soft texture. Unlike regular milk cream, it has the advantage of being able to maintain consistent quality regardless of the production settings. v2foods's soybean-based meat alternatives and those using mushroom mycelium from Meati Foods are examples of meat alternatives.

## Conclusion and Perspectives

The lack of fat and flavor in plant-based meat alternatives is the primary reason for their low popularity. To make meat alternatives more flavorful, further research on fat needs to be conducted. Vegetable oil, mainly MCT oil, is mostly used in vegetable substitute meat products. It is absorbed into the body and mostly used as an instant energy source, making it effective for patients and weight loss. In particular, considering the advantages of vegetable oils, which play a role in reducing the loss of flavor components in the emulsion state, we suggest facilitating more research on vegetable fats. Plant-based meat development is generally focused on improving texture. Recent comparative experiments on soybean powder and differences between various varieties have also been conducted to reduce the distinctive soybean smell in the products and create sensory factors that are closer to those of animal meat. However, it is still difficult to find practical research on how to create the juiciness, taste, and fat flavor unique to animal meat in plant-based meat. Vegetable oil is most commonly used in bakeries, and a study reported that the use of coconut oil can eliminate sensory quality issues [58]. In addition, in the confectionery field, where vegetable oils and proteins are most widely used, it is easy to find research reports showing that their use is less problematic than the use of animal oils or proteins. However, it is often reported that there are still shortcomings in the use of vegetable fats and proteins in the alternative meat market. In fact, research on vegetable proteins is lacking, and research on vegetable fats is even less active. Therefore, we hope that research and government support will be more active to diversify vegetable protein and fat raw materials.

## Futuristic Opinions and Directions

As outlined in the preceding discussion, plant-based alternative foods exhibit promising characteristics such as high protein content, sustainability, and economic viability. Nevertheless, there are several areas that require improvement compared to traditional meat products in terms of similarity. Specifically, plant-based alternatives need enhancements in sensory qualities, including flavor and texture, necessitating developments in additives and processing technologies. However, despite these challenges, plant-based alternatives offer advantages in addressing animal welfare concerns and reducing resource utilization and greenhouse gas emissions. Moreover, they provide a cost-effective source of high-quality protein with verified safety. Additionally, there is potential to achieve unique flavors and aromas distinct from traditional meat by utilizing vegan spices such as turmeric, basil, and coarse pepper. In this context, it is crucial to establish standards for high-value food material production using biotechnology while simultaneously securing new technologies for meat production. This strategic approach is essential to gain a competitive edge in the international landscape of the burgeoning alternative food industry.

## Figures and Tables

**Table 1:** Types of plant-based protein.

Kind	Components	Advantage	Disadvantage	Ref
Soybean	Globulin, albumin, proteose, high lysine and tryptophan	Relatively inexpensive, no scent repulsion	Allergic substances present, GMO, low methionine	[20]
Pea	The most dietary fiber among beans, high lysine and tryptophan	No allergic substances, gluten free, strengthen immunity	Relatively expensive, unique scent	[22]
Wheat	Non-gluten(15%) : Albumin, globulin, protease, / Gluten(85%) : Gliadin, glutenin,	Evenly distributed essential amino acids	Gluten sensitivity leading to digestive problems	[24]
Rice	Glutelin, restricted lysine	Fast digestion and absorption due to small molecular weight	Increasing hardness and chewing to dairy products, Restricted lysine	[38]
Mung bean	Rich leucine, lysine, phenylalanine and tyrosine, Low threonine & tryptophan	Drought-resistant, low-input, gluten free, non GMO detoxification activity, alleviates cardiovascular disease	Allergic substances, difficult cooking	[29]

**Table 2:** Comparison of different production methods of plant-based patties.

Product / Manufacturer	Ingredients	Characteristics
The beyond burger plantbased burger patties / Beyond Meat (America)	Pea protein, expeller-pressed canola oil, refined coconut oil, rice protein, cocoa butter, beet juice color, apple extract etc.	After extracting plant protein, it is mixed with various plant ingredients such as fiber and yeast.
Impossible burger patties made from plants / Impossible Food (America)	Soy protein concentrate, coconut oil, sunflower oil, potato protein, methylcellulose etc.	Use plant-based heme, called leghemoglobin, which is produced by an insertion of DNA from soy plants into genetically engineered yeast.
Heirloom bean veggie burgers / Dr. praeger’s sensible foods (America)	Cooked bean mix (Adzuki beans, pinto beans, great northern beans, yellow eye beans, black eyed peas, red kidney beans), cooked lentils, cremini mushrooms, tomatoes, cooked brown rice, expeller pressed canola oil, potato flakes, carrots, celery, onions, kale, arrowroot powder, spices, sea salt	A collection of beans, lentils, veggies and herbs, gluten free, soy free, vegan certified, and Non-GMO project verified.
Veggie garden V patty / Taekyungnongsan (Republic of Korea)	Soybean protein, vegetable oil, palm oil, grill sauce, fermented apple concentrate, lemon concentrate, high-calorie coloring, oat dietary fiber, etc	High moisture meat analysis (HMMA) method developed independently enables meat-specific juices.
Organic quinoa burger mix / Risenta (Sweden)	Royal quinoa (flakes, grains, flour) 91%, carrot, green kale, parsley, bell pepper, oregano, tomatoes, salt, coriander, garlic, onion, pepper without colorants	The mixture of white royal quinoa and dried vegetables has a high fiber content, suitable for the production of vegan burger patties, without dyes, gluten and lactose-free.
